# Serum concentration–guided intravenous magnesium sulfate administration for neuroprotection in patients with aneurysmal subarachnoid hemorrhage: a retrospective evaluation of a 12-year single-center experience

**DOI:** 10.1007/s10143-023-02159-1

**Published:** 2023-09-26

**Authors:** C. Wipplinger, A. Cattaneo, T. M. Wipplinger, K. Lamllari, F. Semmler, C. Geske, J. Messinger, V. Nickl, A. Beez, R.-I. Ernestus, M. Pham, T. Westermaier, J. Weiland, C. Stetter, E. Kunze

**Affiliations:** 1 Department of Neurosurgery, University Hospital of Würzburg, Würzburg, Germany; 2 Department of Neuroradiology, University Hospital of Würzburg, Würzburg, Germany; 3 Department of Neurosurgery, Helios Amper-Klinikum Dachau, Dachau, Germany

**Keywords:** Aneurysmal subarachnoid hemorrhage, aSAH, Magnesium sulfate, Neuroprotection, Vasospasm, Delayed cerebral infarction

## Abstract

Delayed cerebral infarction (DCI) is a major cause of morbidity and mortality in patients with aneurysmal subarachnoid hemorrhage (aSAH). The benefits of magnesium sulfate as an alternative treatment are controversial, and most previous studies examined its benefits only as adjunctive treatment to traditional nimodipine. We retrospectively analyzed aSAH patients records with magnesium sulfate between 2010 and 2021. We aimed for a serum magnesium concentration of 2–2.5 mmol/l between post-hemorrhage days 3 and 12. The patients were separated in three groups based on average serum magnesium concentration (magnesium >2 mmol/l, reduced magnesium 1.1–1.9 mmol/l, and no magnesium). Additionally, we assessed delayed cerebral infarction (DCI) and clinical outcome at follow-up, using the modified Rankin Scale (mRS), categorized in favorable (0–3) and unfavorable outcome (4–5). In this analysis, 548 patients were included. Hereof, radiological evidence of DCI could be found in 23.0% (*n* = 126) of patients. DCI rates were lower if patients’ average serum magnesium was higher than 2 mmol/l (magnesium 18.8%, *n* = 85; reduced magnesium 38.3%, *n* = 23; no magnesium 51.4%, *n* = 18; *p* < 0.001). Also, at the last follow-up, patients in the group with a higher serum magnesium concentration had better outcome (favorable outcome: magnesium 64.7%, *n* = 293; reduced magnesium 50.0%, *n* = 30; no magnesium 34.3%, *n* = 12; *p* < 0.001). This 12-year study reveals the value of serum concentration-guided magnesium administration in aSAH patients. Our findings demonstrate the safety and efficacy when titrated to a serum concentration of 2–2.5 mmol/l. We observed higher rates of delayed cerebral infarction and unfavorable outcomes in patients with serum concentrations below 2 mmol/l.

## Introduction

Aneurysmal subarachnoid hemorrhage (aSAH) is a severe condition with high morbidity and mortality, representing about 5% of all strokes [1]. Despite recent advancements in the treatment of ruptured intracranial aneurysms, mortality remains substantial. Up to 50% of aSAH patients die in the first few months after bleeding [2, 3], and a significant number of patients who survive must cope with life-altering disability and neurological deficits [3, 4]. Patients with a more severe hemorrhage, classified as Hunt & Hess (H&H) grade 4 and 5, have a poor prognosis despite the development of various treatment modalities and more sophisticated intensive care management [5].

Vasospasm and delayed cerebral ischemia are the most relevant in-hospital risk factors for unfavorable outcome following aSAH. Vasospasm is among the most commonly known causes for cerebral infarction in aSAH patients [6]. Most frequently occurring between days 4 and 14 after hemorrhage, vasospasm has an overall estimated incidence as high as 70% [7]. Delayed cerebral infarction (DCI) has a prevalence between 19% and 35% [6, 8–10]. Previously, DCI was believed to be caused exclusively by angiographically detectable spasms of larger brain vessels. In recent years, however, it is increasingly clear that a complex chain of events contribute to DCI [11, 12].

Traditionally, the oral calcium antagonist nimodipine has been utilized to reduce both the risk for unfavorable outcome and delayed cerebral ischemia [13]. However, as an alternative, magnesium sulfate has been used as prophylactic treatment for aSAH patients [14]. As a physiological calcium antagonist, magnesium is known to be a neuroprotective agent. Additionally, by acting as an N-type calcium channel blocker, which are known to play a role in arterial smooth muscle contraction, magnesium has potential benefits against vasospasm [15–17].

In our institution, we have studied the effects of magnesium sulfate in aSAH patients extensively in experimental as well as in clinical studies [14, 18, 19]. Unlike several multicentric studies that administered magnesium in a fixed-dose manner and failed to find beneficial effects [20], a randomized controlled trial in our institution demonstrated the effectiveness of a tailored magnesium therapy guided by serum concentration. After the successful results of this trial, we implemented magnesium sulfate as a routine treatment in aSAH patients. The present study now aims to retrospectively analyze the outcomes of this serum guided treatment, assessing its impact as a routine therapy over a 12-year period.

## Methods

### Institutional standard management of aSAH patients

In our institution, all aSAH patients are monitored in the intensive care unit (ICU) for a minimum of 12 days unless they are transferred to a different hospital earlier. Aneurysm treatment is performed within 48 h whenever possible. After aneurysm treatment, we maintain euvolemia and aim for mild hypertension with a target mean arterial pressure of 80 mmHg. Vasospasm monitoring involves transcranial doppler (TCD) sonography and neurological examinations in awake patients twice a day. Vasospasm on TCD is defined as mean flow velocity over 140 cm/s in the anterior circulation or 90 cm/s in the basilar artery or an increase of more than 30 cm/s within 24 h. Should a patient display symptoms or TCD suggest vasospasm, a digital subtraction angiography (DSA) is performed with the goal of vasospasmolysis, employing intraarterial nimodipine infusion, balloon angioplasty, or a hybrid technique, as needed.

For a more detailed description of our institutional management of aSAH patients, we refer to our previous works [14, 18, 21].

### Magnesium sulfate application

Continuous intravenous magnesium is started between day 0 and 1, immediately after aneurysm treatment, with an initial rate of 8 mmol/h. Over the first 3 to 4 days after hemorrhage, dose adjustments are made until a target serum concentration of 2–2.5 mmol/l is achieved. This serum concentration is then maintained until days 12–14 following aSAH, or until day 20 in case of ongoing vasospasm [14, 18, 19]. Serum magnesium concentrations are checked every 8 h. Should side effects like bradycardia or hypotension occur, the dosage of magnesium sulfate is either temporarily discontinued or reduced until symptoms resolve. Magnesium treatment is then resumed at an appropriate dosage.

### Imaging analysis

In patients under continuous sedation, routine CT scans are obtained on days 3 or 4, 6 or 7, 9 or 10 post aSAH, and before discharge. If awake patients are amenable for neurological examination, CT or MRI scans are only performed in case of clinical deterioration, or if EVD problems occur.

Hypodensities on brain CT scans were classified as follows: (1) preexisting; (2) exclusively resulting from intracerebral hematoma; (3) caused by operative procedures; or (4) delayed cerebral infarction defined as hypodensities on CT or respective findings in MRI appearing between day 3 and the end of the observation period after exclusion of procedure related infarctions [22]. Angiographic vasospasm was defined as narrowing of the arterial diameter of >30% in DSA with significant delay of circulation time.

### Variables and measurements

The primary outcome variable for our study was DCI, defined as proposed by Vergouwen et al. [22]. Patients were separated in three groups based on their average serum magnesium concentration between days 3 and 12, namely *magnesium* group (≥2 mmol/l), *reduced magnesium* dose group (1.1–1.9 mmol/l), and *no magnesium* group (<1.1 mmol/l). Additionally, we evaluated the Glasgow Coma Scale on admission as well as the aSAH severity by means of the H&H score. The location of the aneurysm identified as the most likely bleeding source was separated in two groups (anterior and posterior circulation). The aSAH pattern was graded according to the modified Fisher Scale.

We recorded interventions for vasospasm (i.e., intraarterial administration of nimodipine or balloon angioplasty).

The functional outcome at discharge and at the last clinical follow-up visit after discharge was defined using the modified Rankin scale (mRS).

### Patient sample and study design

Data from aSAH patients who were admitted our department between January 2010 and December 2021 were retrospectively reviewed, anonymized, and analyzed. The study was conducted in accordance with the Declaration of Helsinki and approved by our institutional ethics board.

We included patients aged 18 and above with an aSAH caused by aneurysms with complete medical records. Patients with aSAH from any causes other than an intracranial aneurysm were excluded from this analysis. Since our primary outcome measure was DCI, by definition, occurring earliest on day 3, we excluded patients who died within 3 days. Patients who spent less than 12 days in our ICU for any reason other than demise (i.e., transfer to a different hospital) were excluded from the analysis.

### Statistical analysis and data collection

Data were extracted from our institutional general patient data management system SAP (SAP AG, Wallendorf, Germany).

The Kolmogorov-Smirnov test was used to determine normal distribution. Normally distributed data were expressed as mean ± standard deviation and skewed data as median and interquartile range with the 25th and 75th percentiles.

Relationships between categorical variables were determined by the chi-square test. The Mann-Whitney *U* test was used to compare differences between continuous and nominal variables. A *p*-value <0.05 was considered statistically significant, and all *p*-values were two-tailed.

To determine the overall effect of magnesium on the development of DCI, average serum magnesium concentrations between days 3 and 12 were incorporated into a binary logistic regression analysis along with potential confounding factors, patient specific demographics, treatment modalities, comorbidities, and the presence of vasospasm.

Additionally, to account for the variability in follow-up periods, a Cox proportional-hazards regression model was used to identify factors significantly influencing clinical outcomes.

All statistical evaluations were performed with SPSS Version 28.0 (IBM Corp. Released 2021. IBM SPSS Statistics for Mac OS X, Version 28.0, NY: IBM Corp.).

## Results

We identified 974 patients treated for subarachnoid hemorrhage in our department between January 2010 and December 2021. We excluded 426 patients for the following reasons: 98 patients were excluded as they were admitted more than 96 h after hemorrhage, 26 patients because they received aneurysm treatment in a different hospital before being transferred to our institution, 27 patients experienced demise within 3 days, 177 patients had no detectable source of hemorrhage, 46 patients had a bleeding source other than an intracranial aneurysm, 31 patients had incomplete records, and 21 patients were excluded because they received treatment for less than 14 days in our institution. Ultimately, 548 patients met all inclusion criteria and were included in this retrospective analysis.

### Magnesium sulfate administration

In 453 patients (82.7%), an average serum magnesium concentration of 2.1 mmol/l could be maintained between days 3 and 12 (*magnesium* group).

In 60 patients (11.0%), side effects including hypotension and bradycardia occurred and the continuous magnesium dose was reduced until they resolved. In this group (*reduced magnesium* group), the average serum magnesium concentration between days 3 and 12 was 1.4 mmol/l.

**Fig. 1:**
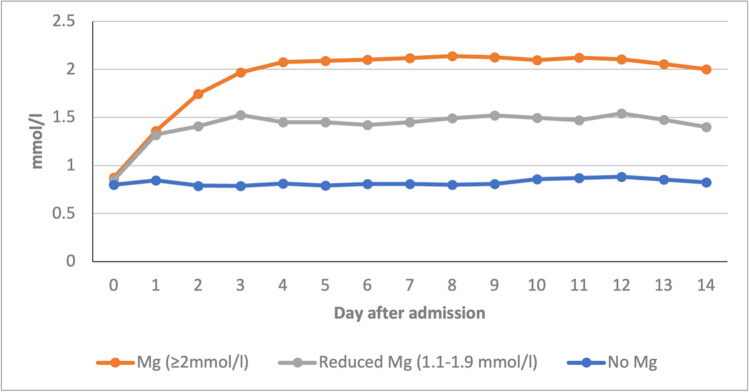
Magnesium concentration of the three groups magnesium, reduced magnesium, and no magnesium over 14 days; Mg, magnesium

In 35 patients (6.3%), magnesium sulfate was not administered (*no magnesium* group) because of preexisting severe kidney failure with a glomerular filtration rate below 20 ml/min or because of a preexisting bradyarrhythmia. In this group, the average serum magnesium concentration between days 3 and 12 was 0.8 mmol/l (Fig. 1). Aside from those of the 60 patients in the reduced magnesium group, no other significant side effects were observed. None of the included patients received oral or intravenous nimodipine. The baseline demographics of the three treatment groups are found in Table 1.

**Table 1:** Baseline characteristics at presentation and initial treatment; *n*, number; *SD*, standard deviation

	Magnesium (≥ 2 mmol/l), *n* = 453	Reduced magnesium (1.1–1.9 mmol/l), *n* = 60	No magnesium, *n* = 35	Overall	*p*-value
Male (*n* and %)	137 (30.2%)	24 (40.0%)	15 (42.9%)	176 (32.1%)	0.117
Female (*n* and %)	316 (69.8%)	36 (60.0%)	20 (57.1%)	372 (67.9%)	
Age at event in years (mean; *SD*)	55.86 ± 12.41	54.87 ± 14.03	54.74 ± 12.78		0.769
Comorbidities (*n* **= **548)					
Smoker (*n* and %)	141 (31.1%)	11 (22%)	11 (31.4%)	163 (29.7%)	0.122
Diabetes (*n* and %)	28 (6.2%)	5 (10%)	0 (0.0%)	33 (6.0%)	0.243
Adipositas (*n* and %)	35 (7.7%)	3 (6.0%)	1 (2.9%)	39 (7.1%)	0.453
Hypertension (*n* and %)	204 (45.0%)	27 (54%)	11 (31.4%)	242 (44.2%)	0.597
Cardiovascular disease (*n* and %)	51 (11.3%)	9 (18%)	5 (14.3%)	65 (11.9%)	0.631
Chronic kidney failure (*n* and %)	35 (7.7%)	2 (4%)	12 (34.3%)	49 (8.9%)	<0.001
Ruptured aneurysm location (*n* = 548)					
Anterior circulation (*n* and %)	374 (82.6%)	49 (81.7%)	32 (91.4%)	455 (83.0%)	0.388
Posterior circulation (*n* and %)	79 (17.4%)	11 (18.3%)	3 (8.6%)	93 (17.0%)	
Aneurysm treatment modality (*n* = 548)					
Surgical treatment (*n* and %)	146 (32.2%)	23 (38.3%)	8 (22.9%)	177 (32.3%)	0.167
Endovascular treatment (*n* and %)	292 (64.5%)	36 (60.0%)	26 (74.3%)	354 (64.6%)	
Conservative treatment (*n* and %)	15 (3.3%)	1 (1.7%)	1 (2.8%)	17 (3.1%)	
Hunt and Hess Grading (*n* = 548)					
1	79 (17.4%)	14 (23.3%)	6 (17.1%)	100 (18.2%)	0.763
2	105 (23.2%)	16 (26.7%)	8 (22.9%)	130 (23.7%)	
3	97 (21.4%)	10 (16.7%)	4 (11.4%)	110 (20.1%)	
4	71 (15.7%)	8 (13.3%)	6 (17.1%)	84 (15.3%)	
5	101 (22.3%)	12 (20.0%)	11 (31.4%)	124 (22.6%)	
Fisher Scale (*n* = 548)					
I	12 (2.7%)	2 (3.3%)	0 (0.0%)	14 (2.6%)	0.212
II	21 (4.6%)	2 (3.3%)	2 (5.7%)	25 (4.6%)	
III	282 (62.3%)	46 (76.7%)	27 (77.1%)	355 (64.8%)	
IV	138 (30.4%)	10 (16.7%)	6 (17.1%)	154 (28.1%)	

### Interventions

The overall most common aneurysm treatment modality in our sample was endovascular. A total of 354 patients (64.6%) received endovascular treatment, while 177 (32.3%) were treated surgically, and 17 (3.1%) conservatively. Conservative treatment was mostly chosen if the complex configuration of the aneurysm required stent placement with a dual antiplatelet regimen. In those cases, treatment was delayed until the acute aSAH phase had passed. Another reason for treating the aneurysm conservatively was due to patient wish. A detailed analysis of the treatment modalities in the respective groups is shown in Table 1.

In our cohort, 46% (*n* = 252) of patients developed vasospasm detectable with DSA. The rate of vasospasm in the regular magnesium treatment group (i.e., serum concentration >2 mmol/l) was slightly higher (*n* = 216, 47.7%) than in the reduced magnesium (*n* = 21, 35.0%) and the no magnesium group (*n* = 15, 42.9%). This difference was not statistically significant (*p* = 0.164).

Among the patients with vasospasm, all but 11 patients underwent an intervention to treat the condition. More than half of those patients (*n* = 138, 57.3%) were treated with balloon angioplasty, while 103 (42.7%) received intraarterial nimodipine only as vasospasm treatment. On average, every patient was treated with two (SD 1.6) interventions; 519 interventions were performed in our cohort (Table 2).

**Table 2:** Total occurrence and type of intervention for vasospasm; *n*, number; *SD*, standard deviation; TCD, transcranial doppler sonography

	Magnesium (≥2 mmol/l)	% or *SD*	Reduced magnesium (1.1–1.9 mmol/l)	% or *SD*	No magnesium	% or *SD*	Total	% or *SD*	*p*-value
TCD vasospasm (*n* and %)	216	48%	21	35%	15	43%	252	46%	0.164
Patients undergoing intervention for vasospasm	208	46%	21	35%	13	37%	241	44%	0.228
*Intra-aerterial nimodipine only (n and %)*	*89*	*43%*	*9*	*43%*	*5*	*38%*	*103*	*43%*	0.247
*Balloon angioplasty (n and %)*	*119*	*57%*	*12*	*57%*	*8*	*62%*	*138*	*57%*	
Total interventions for vasospasm (*n*)	455		38		23		519		
Average number of interventions per patient (average and *SD*)	2.19	1.75	2	1.3	1.8	1.2	2	1.6	0.233

**Fig. 2:**
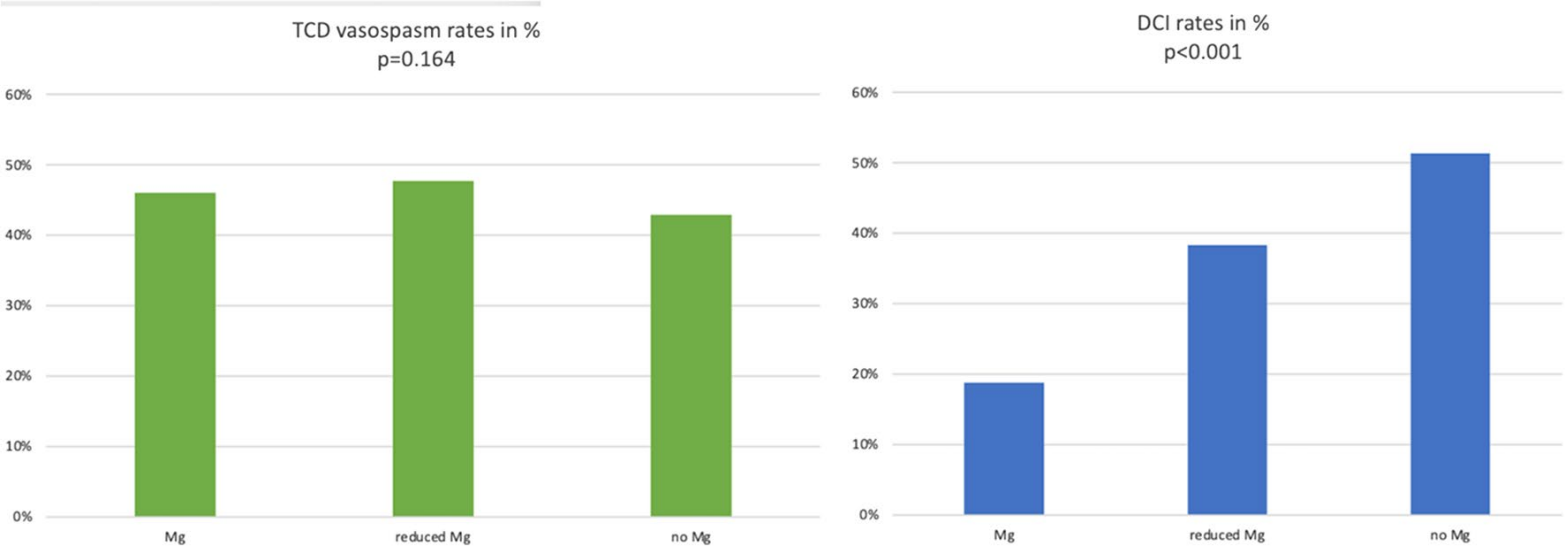
TCD vasospasm rates in percent (left, *p* = 0.164) and DCI rates in percent (right, *p* < 0.001) of the three groups; DCI, delayed cerebral infarction; Mg, magnesium; TCD, transcranial doppler sonography

Overall, radiological evidence of DCI was found in 23.0% (*n* = 126) patients, of which 79.3% (*n* = 100) had vasospasm (*p* < 0.001). The magnesium group had significantly lower rates of DCI (18.8%) than the reduced magnesium (38.3%) and the no magnesium group (51.4%) (*p* < 0.001; Fig. 2).

In a binary logistic regression analysis, we identified factors that influenced the occurrence of DCI while controlling for potential confounders. As depicted in Table 3, magnesium concentration was strongly associated with a reduced risk of DCI (OR 0.21, 95% CI 0.111–0.398, *p* < 0.001). The only other significant predictor for the occurrence of DCI was the presence of vasospasm (OR 14.204, *p* < 0.001)

**Table 3:** Results from the binary logistic regression analysis aimed at identifying factors influencing the development of DCI while accounting for potential confounding factors; CI, confidence interval; DCI, delayed cerebral infarction; H&H, Hunt and Hess

Variable	Odds ratio	95% CI	*p*-value
Serum magnesium concentration between day 3 and 12	**0.21**	**0.111–0.398**	**<0.001**
Vasospasm	**14.204**	**6.926–29.131**	**<0.001**
Smoker	0.6	0.300–1.198	0.148
Diabetes	0.375	0.070–1.993	0.25
Adipositas	3.283	0.853–12.628	0.084
Hypertension	0.555	0.300–1.026	0.06
Cardiovascular disease	0.923	0.342–2.491	0.875
Chronic kidney failure	1.046	0.425–2.578	0.922
H&H 4–5	0.999	0.817–1.222	0.993
Gender	1.248	0.651–2.391	0.505
Surgical aneurysm treatment	0.967	0.517–1.808	0.916
Posterior circulation	0.704	0.306–1.620	0.409
Age at event	1.016	0.988–1.045	0.268

The bold emphasis means that this value is statistically significant

### Outcome

The average follow-up time was 11.9 months. The longest follow-up was 142 months; some patients did not return after discharge, resulting in a follow-up time of under 1 month.

At their last recorded follow-up, 335 patients had an mRS of 0–3, 117 patients 4–5, and 96 were deceased. In the magnesium group, 293 patients (64.7%) had an mRS of 0–3, 82 (18.1%) an mRS of 4–5, and 78 (17.2%) were deceased.

In the reduced magnesium group, 30 patients (50.0%) had an mRS of 0–3, 22 (36.7%) had an mRS of 4–5, and 8 (13.3%) died in the meantime.

**Fig. 3:**
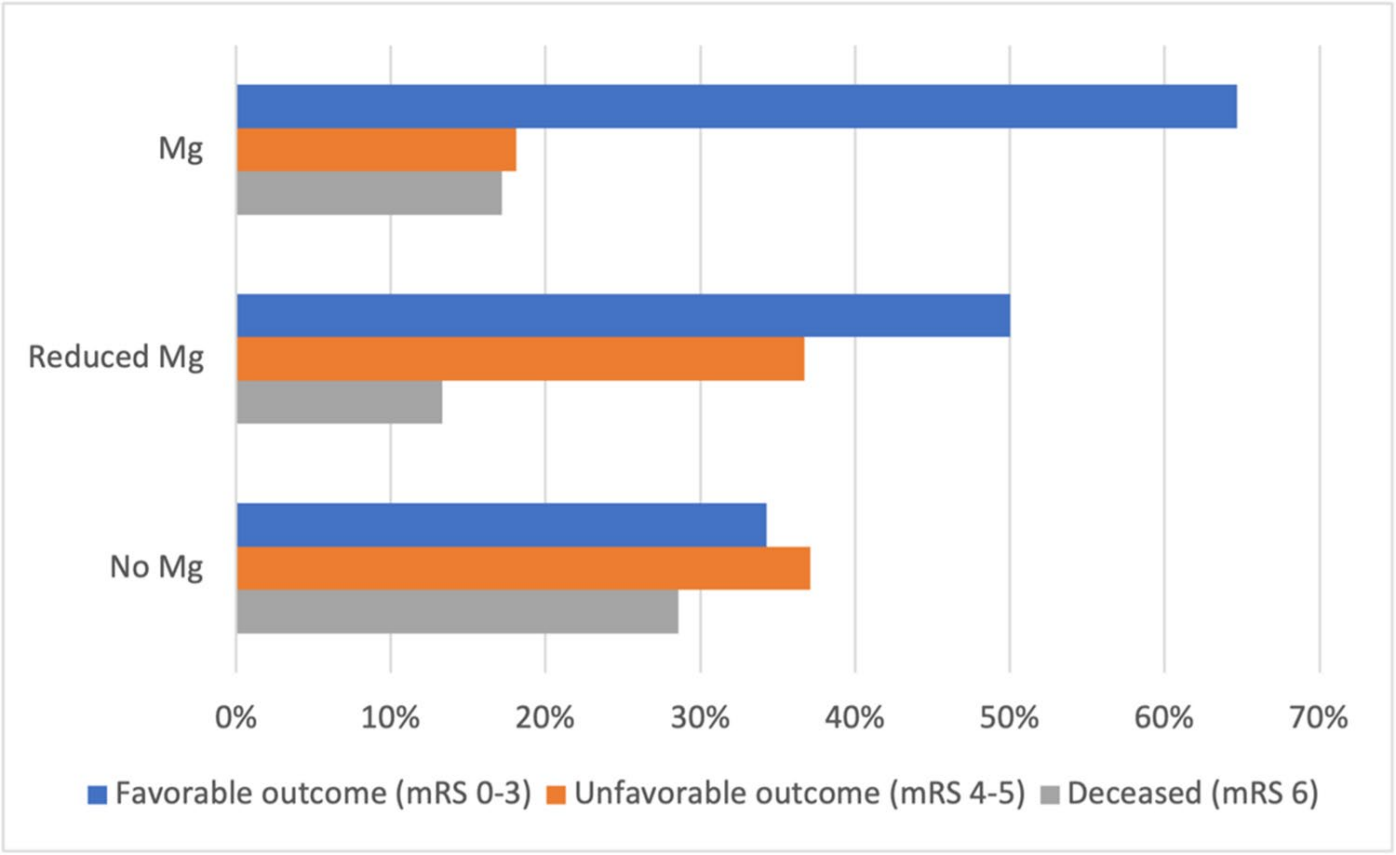
Functional outcome of the three groups, *p* < 0.001; Mg, magnesium; mRS, modified Rankin Scale

Among patients who did not receive magnesium, 12 patients (34.3%) had an mRS of 0–3, 13 (37.1%) had an mRS of 4–5, and 10 (28.6%) had died (*p* < 0.001; Fig. 3).

**Fig. 4:**
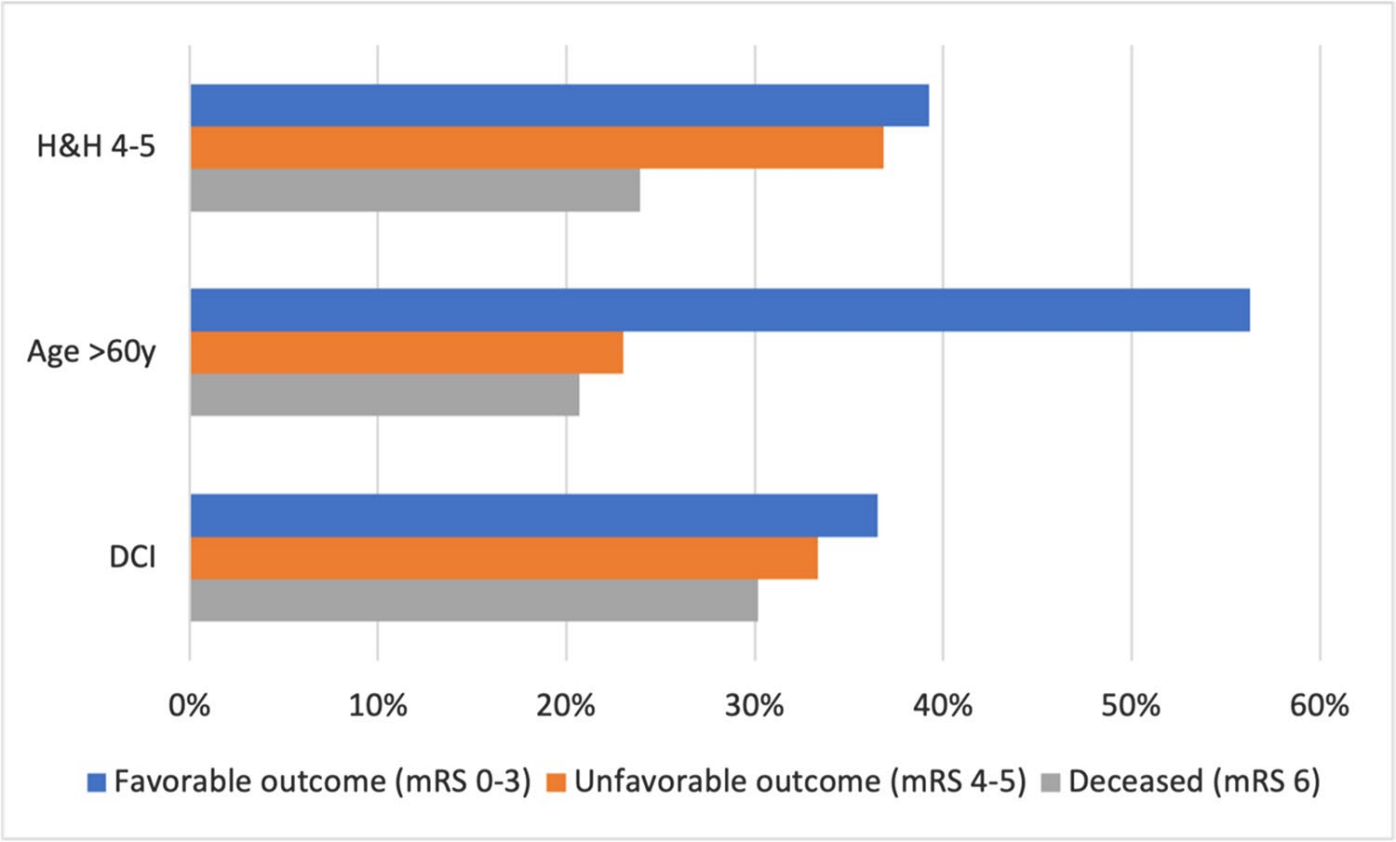
Significant predictors for unfavorable outcome/death; DCI, delayed cerebral infarction; H&H, Hunt and Hess; mRS, modified Rankin Scale; y, years

To account for the high variability in follow-up times, a Cox proportional hazards regression model was used as highlighted in Table 4. We identified that higher serum magnesium concentrations significantly reduced the risk for unfavorable outcome/death (HR 0.66, 95% CI 0.479–0.909, *p* = 0.011). Risk factors contributing to unfavorable outcome/death included increased age, H&H grades 4–5, and the presence of DCI (Fig. 4).

**Table 4:** Results from a Cox proportional-hazards model identifying factors that influenced the overall outcomes while accounting for the variability in follow-up times; CI, confidence interval; DCI, delayed cerebral infarction; H&H, Hunt and Hess

Variable	Hazard Ratio	95% CI	p-value
Serum magnesium concentration between days 3 and 12	**0.66**	**0.479–0.909**	**0.011**
H&H 4–5	**2.244**	**1.585–3.178**	**<0.001**
DCI	**2.042**	**1.340–3.113**	**<0.001**
Age at event	**1.032**	**1.015–1.048**	**<0.001**
Smoker	0.866	0.565–1.328	0.51
Diabetes	1.096	0.517–2.321	0.811
Adipositas	1.311	0.623–2.757	0.475
Hypertension	0.997	0.704–1.410	0.985
Cardiovascular disease	1.157	0.705–1.897	0.564
Chronic kidney failure	1.113	0.674–1.840	0.675
Vasospasm	0.89	0.600–1.320	0.562
Gender	1.127	0.780–1.628	0.524
Surgical aneurysm treatment	0.846	0.591–1.212	0.362
Posterior circulation	1.195	0.727–1.964	0.483

The bold emphasis means that this value is statistically significant

## Discussion

The present study provides a 12-year single-center experience with magnesium sulfate administered in a serum guided fashion as neuroprotective treatment for aSAH patients. The findings of the present study corroborate the results of our previous randomized controlled trial. Notably, we found that only 11% of patients experienced side effects that prompted us to reduce the magnesium dose and only 6% had comorbidities that prohibited magnesium administration. Additionally, we observed significantly higher DCI rates if the minimum target concentration of 2 mmol/l could not be achieved.

### Dose and serum concentration

The current guidelines for the management of aSAH patients do not support the routine use of magnesium [23]. After publication of several multicentric randomized controlled trials, the use of magnesium was largely abandoned. Among the most prominent of these studies was the MASH-2 trial, an elaborately designed multicentric randomized controlled trial with a large sample size that found no significant difference in functional outcomes. Similar conclusions can be found in several systematic reviews and meta-analyses [4, 20, 24]. However, MASH-2, like many others, administered a fixed dose of 64 mmol/day magnesium sulfate and nimodipine, while the control group received a placebo and nimodipine. Since both nimodipine and magnesium sulfate act as calcium antagonists, their concomitant use is unlikely to yield additional benefits. In another randomized controlled trial, Wong et al. adjusted the dose to achieve approximately twice the baseline serum concentration with a maximum serum concentration of 2.5 mmol/l. Overall, the average serum magnesium concentration in the treatment group was 1.67 mmol/l, similar to our reduced magnesium group. The authors reported no clinical benefit of magnesium sulfate. However, similar to MASH-2, Wong et al. also compared magnesium and nimodipine with placebo and nimodipine [25].

An overview of previous studies investigating the neuroprotective effects of magnesium sulfate in aSAH patients is provided in Table 5.

**Table 5:** Overview of previous studies investigating the neuroprotective effects of magnesium sulfate on patients with aSAH; aSAH, aneurysmal subarachnoid hemorrhage; d, day; DCI, delayed cerebral infarction; GOS, Glasgow Outcome Scale; iv, intravenous; Mg, magnesium; PO, perioral

Author	Year	Treatment	Control	Start dose	Dose	Duration	Results
Dorhout et al. [26]	2012	iv Mg + nimodipine	Placebo + nimodipine	-	64 mmol/d	20 d	No significant difference in outcome between groups
Hassan et al. [27]	2011	Mg + nimodipine	nimodipine	16 mmol	65 mmol/d	14 d	No significant difference between groups but tendency in the Mg group to have better outcomes
Huenges et al. [28]	2018	Mg	Placebo	-	64 mmol/d	20 d	Treatment with Mg has no effect on cognitive outcome after aSAH.
Veyna et al. [29]	2002	iv Mg + nimodipine	Placebo + nimodipine	-	serum level 1.6–2.3 mmol/l	10 d	A higher percentage of patients (not significant) obtained GOS scores of 4 or 5 in the group treated with Mg
Wong et al. [30]	2006	iv Mg	Placebo	20 mmol	80 mmol/d	14 d	No significant difference between groups in functional recovery or GOS
Wong et al. [25]	2011	iv Mg + nimodipine	Placebo + nimodipine	20 mmol	twice the baseline serum level, maximum 2.5 mmol/l	14 d	No significant difference
van den Bergh et al. [31]	2005	iv Mg + nimodipine	Placebo + nimodipine	-	64 mmol/d	14 d	Mg reduced DCI and prevented poor outcome
Akdemir et al. [32]	2009	iv Mg + placebo	Placebo	20 mmol	64 mmol/d	10 d	Better functional outcomes, no effect against vasospasm
Kunze et al. [18]	2018	Mg	Placebo	16 mmol	192 mmol/d (then serum level 2.0–2.5 mmol)	10 d	Treatment may reduce the risk to develop infarction.
Muroi et al. [33]	2007	iv Mg	Placebo	16 mmol	64 mmol/d	12 d	High-dose Mg therapy might be efficient as a prophylactic adjacent therapy after aSAH to reduce the risk for poor outcome
Prevedello et al. [34]	2005	Mg + nimodipine	Nimodipine	20 mmol	100 mmol/d + increasing in 10–20 mmol/d until 2x serum baseline level		No effect on vasospasm, reduced morbidity, and length of hospitalization
Schmid-Elsässer et al. [35]	2005	Mg	Nimodipine	0.4 mmol/kg	1.2 mmol/kg/d	7 d iv, then up to 21 d PO	Comparably effective to nimodipine in preventing delayed ischemic neurologic deficits
Westermaier et al. [14]	2010	iv Mg	Control	16 mmol	192 mmol/d (then serum level 2.0–2.5 mmol)	10 d	Effective against delayed cerebral infarction, improves outcomes
Current study	**2023**	**iv Mg with serum concentration of 2.0–2.5 mmol/l**	**Patients with serum concentrations <2.0 mmol/l**	**16 mmol**	**192 mmol/d (then serum level 2.0–2.5 mmol)**	**10 d or until resolution of vasospasm**	**DCI rate of 18.8% in patients with ≥2.0 mmol/l, statistically significant difference in outcome**

The bold emphasis means that this value is statistically significant

Administering uniform doses of magnesium neglects individual patient factors such as age, weight, and kidney function [36]. Since magnesium does not readily cross the blood brain barrier, sufficient serum concentrations are required to achieve an effect in the central nervous system [37]. This hypothesis is supported by the findings from Takeuchi et al. [38]. The authors of this study administered magnesium sulfate between days 1 and 14 directly into the basal cisterns, bypassing the blood brain barrier. This led to significantly higher cerebrospinal fluid, and patients were significantly less likely to have cerebral vasospasm or delayed cerebral ischemia with better functional outcomes [38].

### Delayed cerebral infarction

Our primary outcome variable was the development of DCI, which was found in 23.0% (*n* = 126) overall and in 18.8% (*n* = 85) in the magnesium group. We found that DCI had a significant correlation with unfavorable outcome or death and was more common in patients who had magnesium serum concentrations <2 mmol/l (*n* = 53 and 56.0%).

We deliberately chose to adhere to the term “delayed cerebral infarction” given that a significant part of our sample was under general anesthesia during the entire course of their ICU treatment. Thus, clinical aspects of delayed cerebral ischemia could not be recorded. Similarly, several authors of studies in the current literature chose to investigate delayed cerebral infarction rather than ischemia and reported rates between 19.0 and 35.0% in aSAH patients [6, 8, 9].

### Vasospasm treatment

We observed a vasospasm rate of 46.0% (*n* = 252), and, other than in our previous randomized controlled trial, we found no significant difference in patients with serum concentrations <2 mmol/l. Although it did not seem to influence the functional outcome, we could detect a significant association between overall vasospasm rates and DCI.

Earlier studies considered vasospasm as the main risk factor for DCI, but later studies described significant rates of DCI independent of vasospasm [21, 39]. In our study, more than half of the patients with vasospasm did not develop DCI. This may be attributed to the fact that patients with TCD vasospasm received a DSA with subsequent endovascular vasospasm treatment in most cases (Table 2). To date, there are no larger randomized controlled trials investigating the impacts of endovascular treatment for vasospasm. However, several recent retrospective studies confirmed that early endovascular vasospasm treatment improves functional outcomes and may be effective against the development of DCI [40–42].

### Factors influencing outcome

Our Cox proportional hazards regression model revealed that higher serum magnesium concentrations have a significant positive effect on outcomes. Predictors for unfavorable outcomes or death included patient age over 60 years, DCI, and higher H&H grades. While it is a widely accepted fact that DCI and higher grade aSAH are associated with worse outcomes [39], patient age is an increasingly important topic. Katsuki et al. investigated the outcomes of aSAH patients older than 75 years and found that temporal muscle thickness showed a positive correlation with outcomes in elderly patients [43, 44]. As the general life expectancy rises, identifying prognostic markers for the elderly becomes crucial.

### Limitations

The most crucial limitation is the retrospective nature of the current study. Since the three cohorts (i.e., magnesium, reduced magnesium, and no magnesium) were created retrospectively, they are unequal in sample size and lack sufficient statistical power. We attempted to account for confounding factors between groups using multivariate analysis. However, biases may still exist, particularly given that patients in the no magnesium group had significant comorbidities prohibiting them from receiving magnesium treatment as evidenced by the significantly higher proportion of patients with chronic kidney failure. Despite this, we chose to include these patients in our analysis to present a more comprehensive view, recognizing that this may limit the generalizability of the observed intergroup differences. Furthermore, our study’s monocentric character limits the generalizability of our findings. Nevertheless, we found that magnesium sulfate is a safe and effective treatment. It is also worth noting that only a small fraction of our sample was not able to receive a sufficient dose to reach the target serum concentration.

## Conclusion

Our 12-year experience underscores the significance of a tailored approach in magnesium sulfate administration as a neuroprotective treatment in aSAH patients. By titrating to a magnesium serum concentration of 2–2.5 mmol/l, our findings indicate that magnesium is well tolerated. Only a small percentage of patients experienced side effects and an even smaller fraction encountered prohibitive comorbidities.

Additionally, our findings indicate that delayed cerebral infarction and unfavorable clinical outcome rates are higher if a minimum serum concentration of 2 mmol/l is not achieved.

However, while our study provides valuable insights into the benefits of serum concentration–guided magnesium administration, we must acknowledge its limitations due to its retrospective nature.

To draw more definitive conclusions, further multicentric randomized controlled trials investigating the effects of serum concentration–guided magnesium administration in aSAH patients will be necessary.

## Data Availability

Data and materials used for the preparation of this manuscript can be available upon request.
